# Immunogenicity of pembrolizumab in patients with advanced tumors

**DOI:** 10.1186/s40425-019-0663-4

**Published:** 2019-08-08

**Authors:** Marianne J. H. van Vugt, Julie A. Stone, “Rik” H. J. M. M. De Greef, Ellen S. Snyder, Leslie Lipka, David C. Turner, Anne Chain, Mallika Lala, Mengyao Li, Seth H. Robey, Anna G. Kondic, Dinesh De Alwis, Kapil Mayawala, Lokesh Jain, Tomoko Freshwater

**Affiliations:** 1 Integrated Drug Development, Certara, Oss, Netherlands; 2 Quantitative Pharmacology, Merck & Co., Inc, Kenilworth, NJ USA; 3 Decision Analytics, Certara, Princeton, NJ USA

**Keywords:** Immunogenicity, Treatment-emergent ADA, Pembrolizumab, Efficacy, Safety, Advanced tumors

## Abstract

**Background:**

Pembrolizumab is a potent, humanized, monoclonal anti–programmed death 1 antibody that has demonstrated effective antitumor activity and acceptable safety in multiple tumor types. Therapeutic biologics can result in the development of antidrug antibodies (ADAs), which may alter drug clearance and neutralize target binding, potentially reducing drug efficacy; such immunogenicity may also result in infusion reactions, anaphylaxis, and immune complex disorders. Pembrolizumab immunogenicity and its impact on exposure, safety, and efficacy was assessed in this study.

**Patients and methods:**

Pembrolizumab immunogenicity was assessed in 3655 patients with advanced or metastatic cancer treated in 12 clinical studies. Patients with melanoma, non–small cell lung cancer, head and neck squamous cell carcinoma, colorectal cancer, urothelial cancer, and Hodgkin lymphoma were treated with pembrolizumab at 2 mg/kg every 3 weeks, 10 mg/kg every 2 weeks, 10 mg/kg every 3 weeks, or 200 mg every 3 weeks. An additional study involving 496 patients with stage III melanoma treated with 200 mg adjuvant pembrolizumab every 3 weeks after complete resection was analyzed separately.

**Results:**

Of 3655 patients, 2000 were evaluable for immunogenicity analysis, 36 (1.8%) were treatment-emergent (TE) ADA-positive; 9 (0.5%) of these TE-positive patients had antibodies with neutralizing capacity. The presence of pembrolizumab-specific ADAs did not impact pembrolizumab exposure, nor did pembrolizumab immunogenicity affect the incidence of drug-related adverse events (AEs) or infusion-related reactions. There was no clear relationship between the presence of pembrolizumab-specific ADAs and changes in tumor size across treatment regimens. Of the 496 patients treated with pembrolizumab as adjuvant therapy, 495 were evaluable, 17 (3.4%) were TE ADA–positive; none had neutralizing antibodies.

**Conclusions:**

The incidence of TE (neutralizing positive) ADAs against pembrolizumab was low in patients with advanced tumors. Furthermore, immunogenicity did not appear to have any clinically relevant effects on the exposure, safety, or efficacy of pembrolizumab.

**Trial registration:**

ClinicalTrials.gov, NCT01295827 (February 15, 2011), NCT01704287 (October 11, 2012), NCT01866319 (May 31, 2013), NCT01905657 (July 23, 2013), NCT02142738 (May 20, 2014), NCT01848834 (May 8, 2013), NCT02255097 (October 2, 2014), NCT02460198 (June 2, 2015), NCT01953692 (October 1, 2013), NCT02453594 (May 25, 2015), NCT02256436 (October 3, 2014), NCT02335424 (January 9, 2015), NCT02362594 (February 13, 2015).

**Electronic supplementary material:**

The online version of this article (10.1186/s40425-019-0663-4) contains supplementary material, which is available to authorized users.

## Introduction

The expression of the immune checkpoint inhibitor programmed death 1 (PD-1) and its ligands PD-L1 and PD-L2 on tumor cells is known to play a role in immune evasion [1–4], mediating inhibition of the antitumor immune response to allow tumors to grow unchecked. Therefore, PD-1 pathway blockade may render tumors vulnerable to immune surveillance [2, 3]. Pembrolizumab is an immunoglobulin (Ig) G4 kappa monoclonal antibody that specifically targets the immune checkpoint PD-1, blocking its interaction with its ligands. Pembrolizumab was generated by grafting the variable region sequences of a mouse antihuman PD-1 antibody onto a human IgG4-κ isotype framework containing a stabilizing S228P Fc mutation [5]. It is currently approved in more than 80 countries for the treatment of one or more malignancies, including melanoma, non–small cell lung cancer [NSCLC], small cell lung cancer, head and neck squamous cell carcinoma [HNSCC], classical Hodgkin lymphoma [HL], primary mediastinal large B-cell lymphoma, urothelial carcinoma [UC], gastric cancer, cervical cancer, hepatocellular cancer, Merkel cell carcinoma, renal cell carcinoma, and microsatellite instability–high or mismatch repair–deficient solid tumors [6, 7]. Furthermore, pembrolizumab is currently under evaluation for multiple additional solid tumors and hematologic malignancies [8].

Despite the proven efficacy of monoclonal antibodies as drugs, patients may develop antidrug antibodies (ADAs), which have the potential to alter drug clearance and neutralize target binding and can result in reduction or loss of treatment efficacy [9, 10]. Generation of ADAs can also cause potentially serious hypersensitivity reactions, such as anaphylaxis, infusion reactions, and immune complex–mediated diseases [9–12]. The aim of the current study was to evaluate the immunogenicity of pembrolizumab and report the incidence and clinical relevance of ADAs against pembrolizumab across a variety of tumor types.

## Materials and methods

Pembrolizumab immunogenicity was evaluated using serum samples from patients with advanced or metastatic cancer enrolled in 13 clinical studies, 12 were in the nonadjuvant setting (Additional file 6: Table S1) and 1 in the adjuvant setting in melanoma (KEYNOTE-054 study). The phase III KEYNOTE-054 study (ClinicalTrials.gov identifier, NCT02362594) involved patients with high-risk stage IIIA, IIIB, and IIIC melanoma. A variety of advanced tumor types were evaluated (melanoma, NSCLC, HNSCC, colorectal cancer [CRC], UC, and HL). The dose of pembrolizumab was 2 mg/kg every 3 weeks (Q3W), 10 mg/kg every 2 weeks (Q2W), 10 mg/kg Q3W, or 200 mg Q3W. Samples for evaluation of immunogenicity were collected at baseline (0–24 h before the first treatment); prior to the administration of pembrolizumab at different cycles (cycle 2, cycle 4, cycle 8, etc); at the end of treatment; and at 1 month, 3 months, and 6 months of follow-up.

Patients from all studies provided voluntary written informed consent to participate before study start. The studies were conducted in accordance with the protocol, good clinical practice standards, and the Declaration of Helsinki. The protocols and subsequent amendments were approved by the appropriate institutional review board or ethics committee at each participating institution.

### ADA detection

Patient samples were assessed for the presence of pembrolizumab ADAs using a validated electrochemiluminescence immunoassay on the MesoScale Discovery platform. Pembrolizumab has the potential to interfere with the antibody assays at concentrations above the drug tolerance level (DTL) [13, 14]; therefore, an integrated evaluation of anti pembrolizumab antibody results and pembrolizumab serum concentration was created to enable the interpretation of immunogenicity results [13]. A detailed description of the assay is presented in the Additional file 1. Samples were analyzed at vendor 1 (Intertek) or vendor 2 (PPD) using the same internally validated ADA screening assay and the DTL at the respective vendor. The DTL was 25 μg/mL at vendor 1. Subsequently, the assay was further optimized at vendor 2 to increase the DTL to 124 μg/mL. ADA analyses involved a 3-tiered testing approach [13–16] comprising screening (tier 1), confirmation (tier 2), and antibody titer (tier 3) assessments in accordance with applicable guidelines (Additional file 2: Figure S1) [14].

### Neutralizing antibody detection

Neutralizing antibodies (NAbs)—ADAs that block binding of pembrolizumab to PD-1—were assayed using a validated ligand-binding electrochemiluminescence NAb assay. Tier 2–confirmed ADA-positive samples were analyzed for the presence of NAbs to pembrolizumab (Additional file 1 and Additional file 3: Figure S2).

### ADA patient categorization

**Fig. 1:**
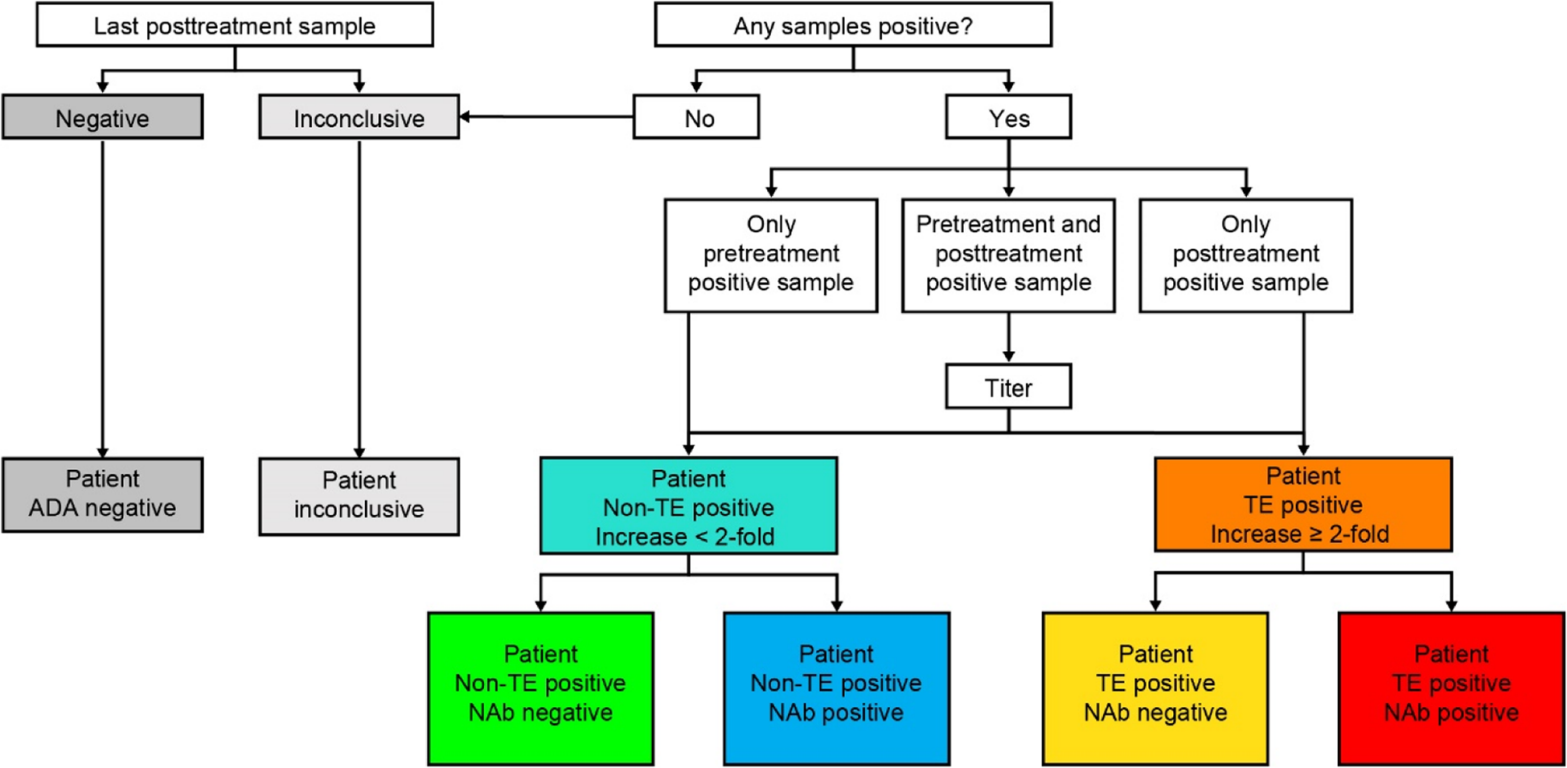
Pembrolizumab ADA patient analysis. *ADA*, antidrug antibody; *NAb*, neutralizing antibody; *non-TE*, non–treatment-emergent; *TE*, treatment-emergent

Immunogenicity of pembrolizumab was evaluated in all assessable patients, ie, all pembrolizumab-treated patients with a pretreatment sample and at least 1 postdose sample, collected after administration of at least 1 pembrolizumab dose. Individual patients were assessed for ADA patient status, composed of 3 categories: inconclusive, negative, and positive [17] as shown in Fig. 1. Patient antidrug antibody status was considered inconclusive if all pretreatment and postdose samples were negative in the confirmatory assay for antipembrolizumab antibodies and the concentration of pembrolizumab in the last postdose sample was above the DTL. A negative ADA patient status was assigned if all pretreatment and postdose samples were negative for antipembrolizumab antibodies in the confirmatory (tier 2) assay and the concentration of pembrolizumab in the last postdose sample was below the DTL (Additional file 2: Figure S1). ADA positivity was confirmed if at least 1 pretreatment or postdose sample was positive in the confirmatory assay for antipembrolizumab antibodies. Patients who were identified as being pembrolizumab ADA-positive were further categorized into treatment-emergent (TE)–positive and non–treatment-emergent (non-TE)–positive patients. For TE-positive patients, the pretreatment sample was negative and at least 1 postdose sample was positive in the confirmatory assay for antipembrolizumab antibody, or both pretreatment and postdose samples were positive with an at least 2-fold increase from baseline. Notably, a more stringent threshold of 2-fold increase in ADA titer from baseline with drug administration was used in the current analysis, compared with the 4- or 9-fold change recommended by Shankar et al. [17]. Conversely, for non-TE–positive patients, the pretreatment sample was positive and all postdose samples were negative in the confirmatory assay for antipembrolizumab antibody or pretreatment and postdose samples were positive with a postdose titer of less than 2-fold of baseline [17]. Both TE-positive and non-TE–positive patients were further classified as NAb negative or NAb positive (Fig. 1).

### Effect of immunogenicity on pembrolizumab exposure, safety, and efficacy

The effect of pembrolizumab immunogenicity on exposure was evaluated by comparing pembrolizumab exposure levels of patients with ADA-positive samples with those of patients receiving the same treatment regimen who had ADA-negative status [18]. The potential impact of pembrolizumab ADAs on drug-related or immune-related adverse events (AEs) was evaluated by comparing the incidence of drug-related or infusion-related reactions in ADA-positive with those in ADA-negative patients. The potential impact of pembrolizumab immunogenicity on efficacy was assessed by comparing the changes in tumor size between ADA-positive and ADA-negative patients.

## Results

### Incidence of immunogenicity of pembrolizumab

A total of 3655 patients with advanced or metastatic cancer who were treated with pembrolizumab were included in the immunogenicity analysis. Of these, 1655 patients had inconclusive results in which all pretreatment and postdose samples were negative in the confirmatory assay for antibodies against pembrolizumab and the concentration of pembrolizumab in the last postdose sample was above the DTL; 2000 patients were evaluable. Of the 2000 evaluable patients, 1943 were negative for ADA and 57 were positive for ADA.

The incidence of TE-positive patients was similar across the dose regimens: 1.5% for 2 mg/kg Q3W, 2.2% for 10 mg/kg Q3W or Q2W, and 1.8% for 200 mg Q3W, resulting in an overall incidence of 1.8% (36 of 2000 evaluable patients) in the pooled analysis of all regimens (Table 1). In the TE ADA–positive patient group, NAbs were detected in 9 of the 36 TE ADA–positive patients (0.5%). Stratification according to tumor type revealed that although the incidence of TE-positive patients was low across indications, there were some differences between some indications. The rates were lowest for HL and melanoma (0.6 and 0.7%, respectively), were highest for NSCLC (2.9%), and were similar for HNSCC (1.6%), CRC (1.9%), and UC (1.4%). The incidence of TE NAb–positive patients was similar for melanoma, NSCLC, HL, and UC (range, 0.2–0.6%), there were no cases with TE Nab–positive patients for either HNSCC or CRC (Table 1).

**Table 1:** Overview of pembrolizumab immunogenicity findings

Pembrolizumab in the nonadjuvant setting: melanoma, NSCLC, HNSCC, CRC, HL, UC						
Stratified by treatment	All treatments	Treatment				
		2 mg/kg Q3W	10 mg/kg Q3W/Q2W	200 mg Q3W		
Immunogenicity status						
Assessable patients^a^	3655	667	2007	981		
Inconclusive patients^b^	1655	125	1462	68		
Evaluable patients^c^	**2000**	**542**	**545**	**913**		
Negative^d^	1943 (97.2%)	527 (97.2%)	529 (97.8%)	887 (97.2%)		
Non–treatment-emergent positive^d^	21 (1.1%)	7 (1.3%)	4 (0.7%)	10 (1.1%)		
Neutralizing negative	19 (1.0%)	5 (0.9%)	4 (0.7%)	10 (1.1%)		
Neutralizing positive	2 (0.1%)	2 (0.4%)				
Treatment-emergent positive^d^	36 (1.8%)	8 (1.5%)	12 (2.2%)	16 (1.8%)		
Neutralizing negative	27 (1.4%)	6 (1.1%)	11 (2.0%)	10 (1.1%)		
Neutralizing positive	9 (0.5%)	2 (0.4%)	1 (0.2%)	6 (0.7%)		
Stratified by indication	Melanoma	NSCLC	HNSCC	CRC	HL	UC
Immunogenicity status						
Assessable patients^a^	1465	1236	101	54	220	579
Inconclusive patients^b^	1063	445	39	0	38	70
Evaluable patients^c^	**402**	**791**	**62**	**54**	**182**	**509**
Negative^d^	395 (98.3%)	762 (96.3%)	59 (95.2%)	51 (94.4%)	179 (98.4%)	497 (97.6%)
Non–treatment emergent positive^d^	4 (1.0%)	6 (0.8%)	2 (3.2%)	2 (3.7%)	2 (1.1%)	5 (1.0%)
Neutralizing negative	3 (0.7%)	5 (0.6%)	2 (3.2%)	2 (3.7%)	2 (1.1%)	5 (1.0%)
Neutralizing positive	1 (0.2%)	1 (0.1%)				
Treatment emergent positive^d^	3 (0.7%)	23 (2.9%)	1 (1.6%)	1 (1.9%)	1 (0.6%)	7 (1.4%)
Neutralizing negative	2 (0.5%)	18 (2.3%)	1 (1.6%)	1 (1.9%)		5 (1.0%)
Neutralizing positive	1 (0.2%)	5 (0.6%)	0	0	1 (0.6%)	2 (0.4%)

*ADA* Antidrug antibody, *CRC* Colorectal carcinoma, *DTL* Drug tolerance level, *HL* Hodgkin lymphoma, *UC* Urothelial cancer, *NSCLC* Non–small cell lung carcinoma, *HNSCC* head and neck squamous cell carcinoma, *NSCLC* Non–small cell lung carcinoma, *Q2W* every 2 weeks, *Q3W* every 3 weeks, *UC* urothelial carcinoma

^a^Included are patients with at least 1 ADA sample available after treatment with pembrolizumab

^b^Inconclusive patients are the number of patients with no positive ADA samples and the drug concentration in the last sample above the DTL

^c^Evaluable patients are the total number of negative and positive patients (non–treatment emergent and treatment emergent)

^d^Denominator was total number of evaluable patients, highlighted in bold

### Effect of pembrolizumab as adjuvant treatment on the immunogenicity incidence rate

In addition, 496 patients with melanoma treated with pembrolizumab as adjuvant therapy in KEYNOTE-054 were analyzed separately. This separate analysis was motivated by an interest in characterizing immunogenicity in a previous line of therapy during which patients would have had less exposure to prior systemic chemotherapy that could impact the status of their immune system. Of these 496 patients treated in the adjuvant setting, 495 were evaluable for the current analysis, 1 patient was inconclusive (Additional file 7: Table S2). At 3.4% (17 of 495, 473 negative, 5 non-TE positive, and 17 TE positive), the incidence of TE ADAs in evaluable patients was numerically higher than for their counterparts treated in the nonadjuvant setting. None of the 17 TE positive patients had antibodies with neutralizing capacity (Additional file 7: Table S2).

### Effect of immunogenicity on pembrolizumab exposure

Overall, pembrolizumab exposure in ADA-positive patients was similar to that in ADA-negative patients at each dose regimen (pembrolizumab at 200 mg Q3W, 2 mg/kg Q3W, 10 mg/kg Q3W, and 10 mg/kg Q2W) (Additional file 4: Figure S3A–D). The presence of NAbs did not appear to influence pembrolizumab exposure. The findings for patients with melanoma treated in the adjuvant setting at a pembrolizumab dose of 200 mg Q3W were similar to those of the pooled indications treated in the nonadjuvant setting at the same dose (Additional file 4: Figure S3A–D and Additional file 5: Figure S4).

### Effect of immunogenicity on safety

Safety was analyzed in the 2000 evaluable patients. Of the 1943 ADA-negative patients, 1338 (68.9%) experienced drug-related AEs (Table 2). Of the 57 ADA-positive patients, of whom 40 (70.2%) experienced drug-related AEs, 36 were TE positive, of whom 27 (75%) had drug-related AEs. The incidence of drug-related AEs was comparable between ADA-negative (68.9%) and TE NAb–positive patients (66.7%). Infusion-related reactions occurred in 43 (2.2%) ADA-negative patients and none of the ADA-positive patients. The small number of TE ADA–positive patients makes any comparison between the incidence of events between the populations difficult.

**Table 2:** Summary of AEs by ADA category after pembrolizumab treatment

	ADA-negative patients	ADA-positive patients, *n* = 57				Total patients
		Non-TE NAb negative	Non-TE NAb positive	TE NAb negative	TE NAb positive	
Patients in population	1943	19	2	27	9	2000
Patients with drug-related^a^ AE, n (%)	1338 (68.9)	11 (57.9)	2 (100)	21 (77.8)	6 (66.7)	1378 (68.9)
Patients with infusion-related reaction,^b^ n (%)	43 (2.2)	0 (0.0)	0 (0.0)	0 (0.0)	0 (0.0)	43 (2.2)

*ADA* Antidrug antibody, *AE* Adverse event, *NAb* Neutralizing antibody, *Non-TE* Non–treatment-emergent ADA positive, *TE* Treatment emergent ADA positive

^a^Determined by the investigator to be related to the drug

^b^Infusion-related reaction is defined as the occurrence of 1 or more of the following preferred terms (MedDRA Version 21) [19]: hypersensitivity, drug hypersensitivity, anaphylactic reaction, anaphylactoid reaction, cytokine release syndrome, serum sickness, serum sickness-like reaction, infusion-related reaction

### Effect of immunogenicity on efficacy

**Fig. 2:**
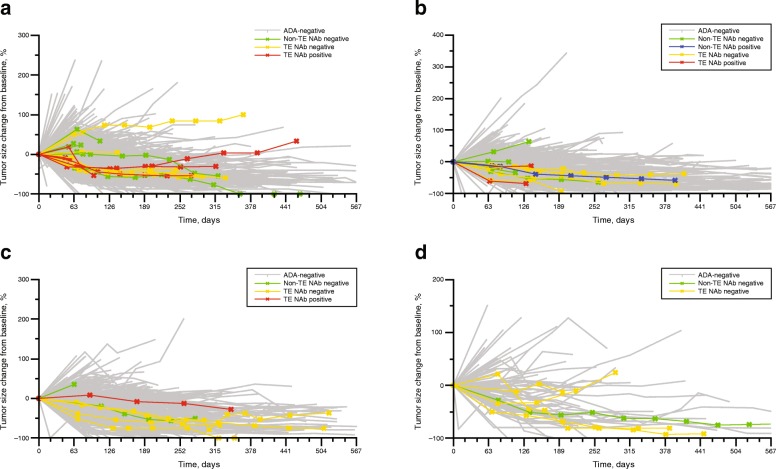
Change in tumor size of evaluable patients treated with pembrolizumab in the non-adjuvant setting at doses of 200 mg Q3W (*N* = 713) (**a**), 2 mg/kg Q3W (*N* = 522) (**b**), 10 mg/kg Q3W (*N* = 399) (**c**), and 10 mg/kg Q2W (*N* = 95) (**d**). Figure includes tumor size change for all ADA evaluable patients. *ADA*, antidrug antibody; *NAb*, neutralizing antibody; *non-TE*, non–treatment-emergent ADA positive; *Q2W*, every 2 weeks; *Q3W*, every 3 weeks; *TE*, treatment-emergent ADA positive

There was no clear relationship between the presence of pembrolizumab-specific ADAs and changes in tumor size across the treatment regimens evaluated (pembrolizumab 200 mg Q3W, 2 mg/kg Q3W, 10 mg/kg Q3W, and 10 mg/kg Q2W; Fig. 2a–d). Similarly, comparable changes in tumor size were observed between patients with and without NAbs.

## Discussion

The current analysis investigated the immunogenicity of pembrolizumab and effects of ADAs on pharmacokinetics, safety, and efficacy in patients with a variety of advanced tumor types, namely melanoma, NSCLC, HNSCC, CRC, UC, and HL. The immunogenicity analysis involved patients treated with pembrolizumab in both the nonadjuvant and adjuvant settings.

The pooled results of this immunogenicity evaluation indicate a low overall incidence of TE ADA positivity (1.8%) and NAb detection (0.5%) after treatment with pembrolizumab in patients in the nonadjuvant setting. These findings were comparable across the 4 different schedules of pembrolizumab treatment studied (200 mg Q3W, 10 mg/kg Q3W or Q2W, and 2 mg/kg Q3W) and in general between the different tumor types. However, there were some numerical differences in rates of TE ADA–positive patients between tumor types, with rates ranging from 0.6 to 0.7% for melanoma and HL to 2.9% for NSCLC. The incidence of TE NAb–positive patients was similar for melanoma, NSCLC, HL, and UC (range, 0.2–0.6%), there were no cases with TE NAb–positive patients for either HNSCC or CRC.

In addition, given the possibility that rates of immunogenicity could differ among patients who have or have not been exposed to prior systemic chemotherapy, which could possibly deplete immune function [20], the immunogenicity of pembrolizumab was also evaluated in a cohort of patients with melanoma treated with pembrolizumab in the earlier adjuvant setting. The incidence of TE pembrolizumab ADAs in patients with melanoma treated in the adjuvant setting was also low. Consistent with the low incidence observed overall in the nonadjuvant setting, there were no cases with NAbs in the adjuvant setting in patients with melanoma (0 of 17).

The presence of pembrolizumab-specific ADAs did not impact pembrolizumab exposure in either the nonadjuvant or the adjuvant settings, suggesting that immunogenicity is unlikely to alter the pharmacokinetics of pembrolizumab. The incidence of drug-related AEs was comparable regardless of ADA status. Although the low incidence of infusion-related reactions limits interpretation, the incidence of infusion-related reactions was lower in ADA-positive (0%) than in ADA-negative (2.2%) patients, suggesting that pembrolizumab immunogenicity did not induce occurrence of infusion-related reactions. Moreover, there was no apparent loss of efficacy, as evidenced by no changes in longitudinal tumor size. However, determination of the impact of immunogenicity on treatment efficacy in this pooled analysis is challenging given the low rate of ADAs and the diverse range of longitudinal tumor size patterns across the various tumor types. With that caveat, the lack of a clear relationship between ADAs and changes in tumor size does suggest that the impact of immunogenicity on efficacy was clinically insignificant.

The immunogenicity assessment for pembrolizumab monotherapy in the nonadjuvant (advanced or metastatic) setting is based on a sufficiently large data set of patients across several indications, with very low observed rates of total TE ADAs (~ 2%) and of NAbs (~ 0.6%) and no demonstrated impact on efficacy or safety, as currently summarized in the United States prescribing information and European Medicines Agency summary of product characteristics for pembrolizumab [6, 7]. The evaluation confirmed the assessment that pembrolizumab has limited potential to elicit the formation of ADAs in the adjuvant monotherapy setting, which is consistent with the results of prior immunogenicity evaluations of pembrolizumab in the nonadjuvant monotherapy setting [6, 21].

In conclusion, the incidence of pembrolizumab ADAs and associated NAbs are low in patients with advanced tumors in both the nonadjuvant and the adjuvant settings. Furthermore, immunogenicity did not appear to have any clinically relevant effects on the exposure, safety, or efficacy of pembrolizumab in either setting.

## Additional files

Additional file 1: Supplementary Methods. (DOCX 21 kb)

Additional file 2: **Figure S1.** Flow chart of ADA sample analysis. ADA, antidrug antibody; DTL, drug tolerance level. (DOCX 220 kb)

Additional file 3: **Figure S2.** Flow chart of neutralizing capacity assessment of ADA-positive samples. ADA, antidrug antibody. (DOCX 264 kb)

Additional file 4: **Figure S3.** Pembrolizumab exposure for patients treated with pembrolizumab in the nonadjuvant setting at doses of 200 mg Q3W (*N* = 913) (a), 2 mg/kg Q3W (*N* = 542) (b), 10 mg/kg Q3W (*N* = 428) (c), 10 mg/kg Q2W (*N* = 117) (d). Figure includes ADA samples with corresponding PK concentrations. Samples taken > 2 times the scheduled time were excluded. Individual pembrolizumab concentrations for the patients are represented as dots or crosses and mean value is represented by a black line. For the positive patients (non-TE and TE), all the samples (ADA negative and ADA positive) are “colored.” The confirmed positive ADA samples are indicated by a black circle around the corresponding PK sample. ADA, antidrug antibody; NAb, neutralizing antibody; non-TE, non–treatment-emergent ADA positive; PK, pharmacokinetic; Q2W, every 2 weeks; Q3W, every 3 weeks; TE, treatment-emergent ADA positive. (DOCX 140 kb)

Additional file 5: **Figure S4.** Pembrolizumab exposure for patients treated with pembrolizumab in the adjuvant setting at a dose of 200 mg Q3W (*N* = 495). Figure includes ADA samples with corresponding PK concentrations. Samples taken > 2 times than the scheduled time were excluded. Individual pembrolizumab concentrations for the patients are represented as dots or crosses and mean value is represented by a black line. ADA, antidrug antibody; NAb, neutralizing antibody; non-TE, non–treatment-emergent ADA positive; PK, pharmacokinetic; Q3W, every 3 weeks; TE, treatment-emergent ADA positive. (DOCX 206 kb)

Additional file 6: **Table S1.** Details of clinical studies^a^ included in the immunogenicity analysis of pembrolizumab. (DOCX 17 kb)

Additional file 7: **Table S2.** Overview of pembrolizumab immunogenicity findings in KEYNOTE-054 (ClinicalTrials.gov Identifier, NCT02362594). (DOCX 13 kb)

## Data Availability

Merck Sharp & Dohme Corp., a subsidiary of Merck & Co., Inc., Kenilworth, NJ, USA’s data sharing policy, including restrictions, is available at http://engagezone.msd.com/ds_documentation.php. Requests for access to the clinical study data can be submitted through the EngageZone site or via email to dataaccess@merck.com

## Abbreviations

ADA: Antidrug antibody
AE: Adverse event
CRC: Colorectal cancer
DTL: Drug tolerance level
ECL: Electrochemiluminescence (supplementary materials only)
HL: Hodgkin lymphoma
HNSCC: Head and neck squamous cell carcinoma
Ig: Immunoglobulin
MSD: MesoScale Discovery (supplementary materials only)
MSI-H: Microsatellite instability–high (supplementary materials only)
NAb: Neutralizing antibody
non-TE: Non–treatment-emergent
NSCLC: Non–small cell lung cancer
PD-1: Programmed death 1
PD-L1: Programmed death ligand 1
PD-L2: Programmed death ligand 2
Q2W: Every 2 weeks
Q3W: Every 3 weeks
TE: Treatment-emergent
UC: Urothelial carcinoma
