# Supplementary material for: Immunogenicity of pembrolizumab in patients with advanced tumors
Source: J Immunother Cancer. 2019 Aug 8;7:212. doi: 10.1186/s40425-019-0663-4 (PMC6686242; doi:10.1186/s40425-019-0663-4)
Supplement: Supplementary file 1 — Supplementary Methods. (DOCX 21 kb) [file 40425_2019_663_MOESM1_ESM.docx]

Additional file 1

**SUPPLEMENTARY METHODS**

**Immunogenicity methods**

Samples were assayed for the presence of pembrolizumab antidrug antibodies (ADAs) using a validated electrochemiluminescence (ECL) immunoassay on the MesoScale Discovery (MSD) platform. Bioanalysis of pembrolizumab ADAs was carried out using the standard 3-tiered assay approach [1-4] that consisted of screening (tier 1), confirmation (tier 2), and antibody titer assessment (tier 3). Only tier 2–confirmed ADA-positive samples were moved to tier 3 and reported with a titer value and a neutralizing antibody (NAb) result.

Pembrolizumab might interfere with the antibody assays at concentrations above the drug tolerance level (DTL). Therefore, an integrated evaluation of anti-pembrolizumab antibody results and pembrolizumab serum concentration was created for interpretation of immunogenicity results. A flow chart of the ADA sample analysis is shown in (supplementary Figure S1).

Tier 2–confirmed ADA-positive samples were also characterized for their neutralizing capacity using a NAb assay based on the ability of ADAs to block (neutralize) the critical first step in the pharmacological action of pembrolizumab, which is binding to programmed death 1 (PD-1) (the *in vivo* target). During the course of the study, measurement of the ADA samples was transferred from 1 vendor (vendor 1, Intertek) to another (vendor 2, PPD). The initial neutralizing assay (vendor 1), was a validated ligand-binding ECL assay and consisted of 2 tiers: a screening tier and a confirmatory tier. The first tier used a cut point aiming for 5% false-positives, while in the second, confirmatory, tier the cut point allowed for 1% false-positives. In the confirmatory tier, protein G depletion was used to confirm the presence of pembrolizumab NAbs (supplementary Figure S2). The neutralizing assay was subsequently redesigned at a second contract research organization (vendor 2). This assay was a validated ligand-binding ECL assay that was adjusted to a single-tier approach. The assay cut point in the first tier aimed for 1% false-positives instead of 5%, supporting the elimination of the second tier. As part of the assay transfer, the ADA screening assay was further optimized to increase the DTL. Additionally, the assay used a purification step, next to the acid dissociation step, to further reduce the influence of remaining drug in the study serum sample. Some of the samples were analyzed at vendor 1 and some of the samples were analyzed at vendor 2. For the evaluation of each individual ADA sample, the DTL of the corresponding location of analysis was used. The DTL for the ADA assay executed at vendor 1 was 25 µg/mL and that for the ADA screening assay executed at vendor 2 was 124 µg/mL.

**References**

1. Mire-Sluis AR, Barrett YC, Devanarayan V, Koren E, Liu H, Maia M, et al. Recommendations for the design and optimization of immunoassays used in the detection of host antibodies against biotechnology products. J Immunol Methods*.* 2004;289(1-2):1-16.

2. Shankar G, Arkin S, Cocea L, Devanarayan V, Kirshner S, Kromminga A, et al. Assessment and reporting of the clinical immunogenicity of therapeutic proteins and peptides-harmonized terminology and tactical recommendations. AAPS J*.* 2014;16(4):658-73.

3. US Department of Health and Human Services, Food and Drug Administration, Center for Drug Evaluation and Research (CDER), Center for Biologics Evaluation and Reseach (CBER). Immunogenicity testing of therapeutic protein products —Developing and validating assays for antidrug antibody detection: guidance for industry. 2019. https://www.fda.gov/media/119788/download. Accessed 9 July 2019.

4. European Medicines Agency. Guideline on immunogenicity assessment of biotechnology-derived therapeutic proteins. 2007. https://www.ema.europa.eu/en/documents/scientific-guideline/guideline-immunogenicity-assessment-biotechnology-derived-therapeutic-proteins-first-version_en.pdf. Accessed July 23, 2019.
