# Supplementary material for: Immunogenicity of pembrolizumab in patients with advanced tumors
Source: J Immunother Cancer. 2019 Aug 8;7:212. doi: 10.1186/s40425-019-0663-4 (PMC6686242; doi:10.1186/s40425-019-0663-4)
Supplement: Supplementary file 2 — Figure S1. Flow chart of ADA sample analysis. ADA, antidrug antibody; DTL, drug tolerance level. (DOCX 220 kb) [file 40425_2019_663_MOESM2_ESM.docx]

Additional file 2: **Figure S1** Flow chart of ADA sample analysis. ADA, antidrug antibody; DTL, drug tolerance level.

**
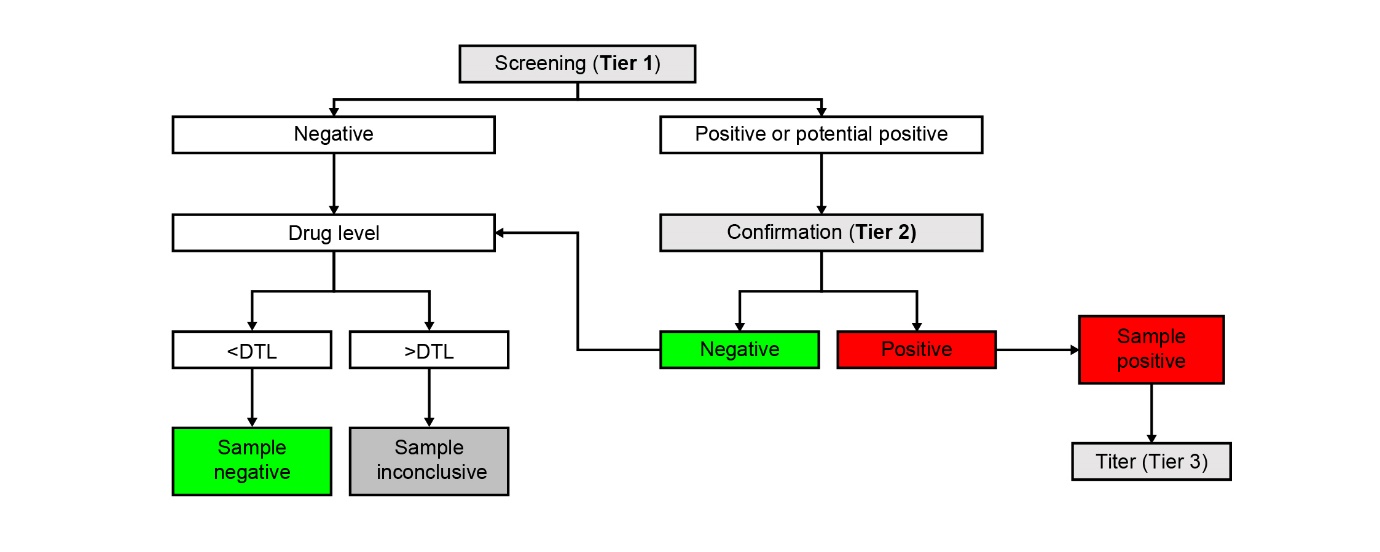
**
