# Supplementary material for: Immunogenicity of pembrolizumab in patients with advanced tumors
Source: J Immunother Cancer. 2019 Aug 8;7:212. doi: 10.1186/s40425-019-0663-4 (PMC6686242; doi:10.1186/s40425-019-0663-4)
Supplement: Supplementary file 3 — Figure S2. Flow chart of neutralizing capacity assessment of ADA-positive samples. ADA, antidrug antibody. (DOCX 264 kb) [file 40425_2019_663_MOESM3_ESM.docx]

Additional file 3: **Figure S2** Flow chart of neutralizing capacity assessment of ADA-positive samples. ADA, antidrug antibody.

**
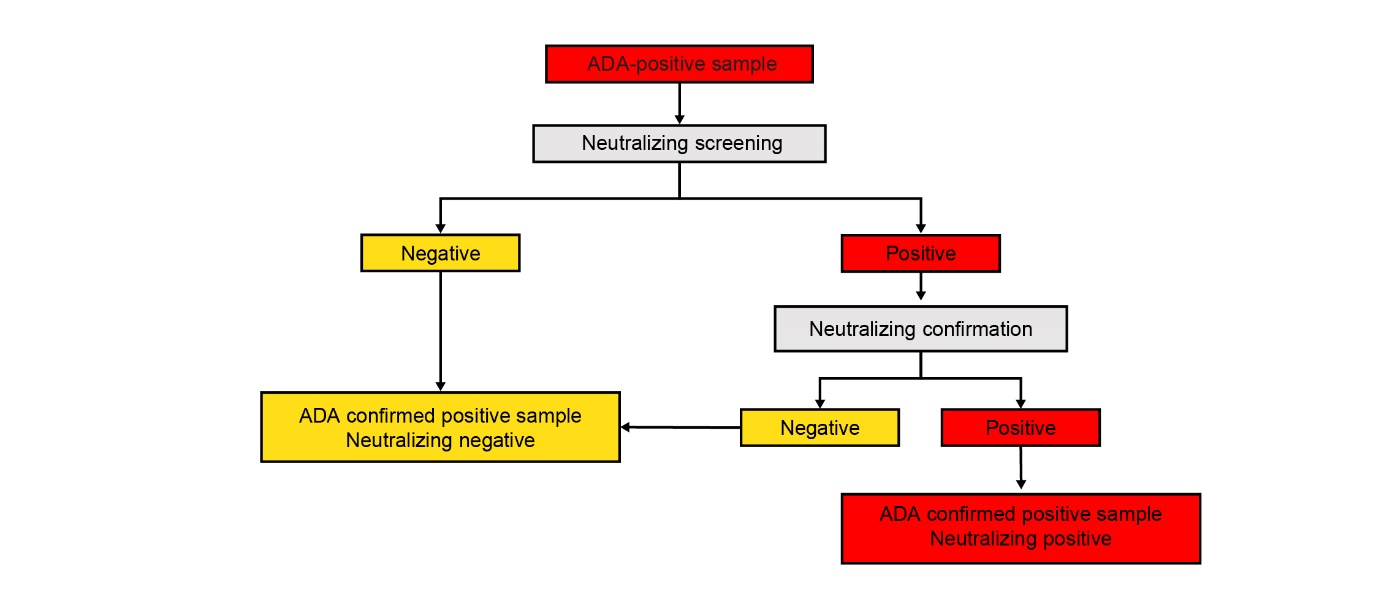
**
