# Supplementary material for: Immunogenicity of pembrolizumab in patients with advanced tumors
Source: J Immunother Cancer. 2019 Aug 8;7:212. doi: 10.1186/s40425-019-0663-4 (PMC6686242; doi:10.1186/s40425-019-0663-4)
Supplement: Supplementary file 4 — Figure S3. Pembrolizumab exposure for patients treated with pembrolizumab in the nonadjuvant setting at doses of 200 mg Q3W (N = 913) (a), 2 mg/kg Q3W (N = 542) (b), 10 mg/kg Q3W (N = 428) (c), 10 mg/kg Q2W (N = 117) (d). Figure includes ADA samples with corresponding PK concentrations. Samples taken > 2 times the scheduled time were excluded. Individual pembrolizumab concentrations for the patients are represented as dots or crosses and mean value is represented by a black line. For the positive patients (non-TE and TE), all the samples (ADA negative and ADA positive) are “colored.” The confirmed positive ADA samples are indicated by a black circle around the corresponding PK sample. ADA, antidrug antibody; NAb, neutralizing antibody; non-TE, non–treatment-emergent ADA positive; PK, pharmacokinetic; Q2W, every 2 weeks; Q3W, every 3 weeks; TE, treatment-emergent ADA positive. (DOCX 140 kb) [file 40425_2019_663_MOESM4_ESM.docx]

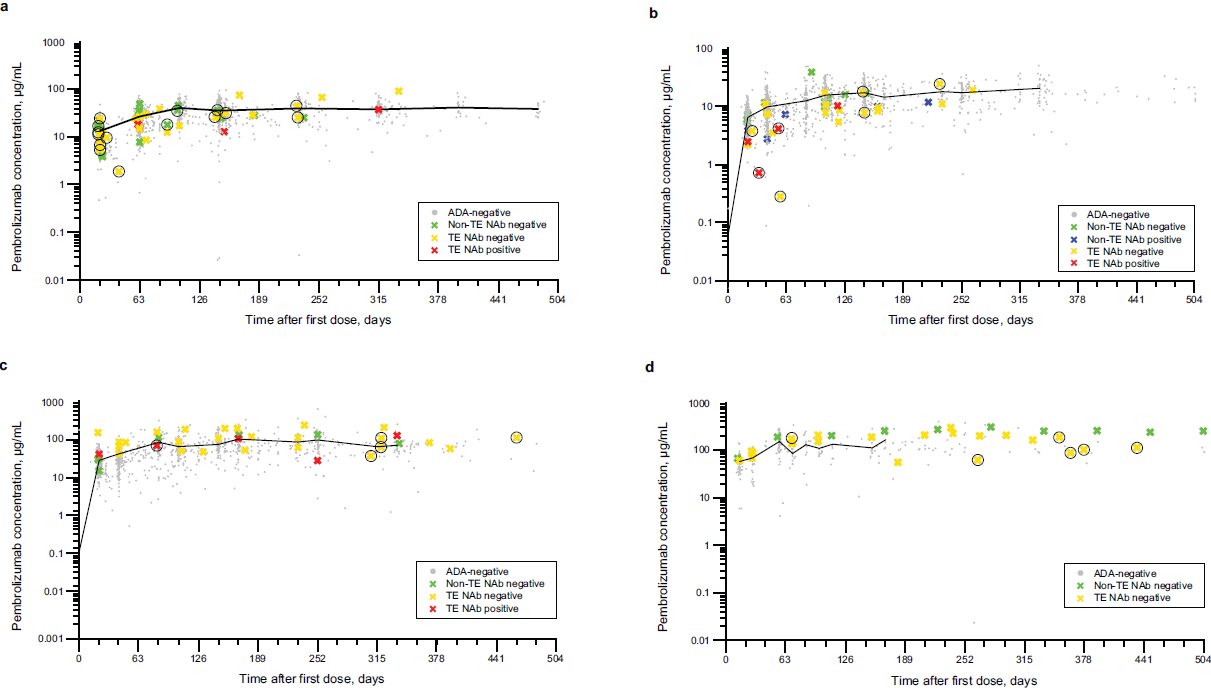
Additional file 4: **Figure S3** Pembrolizumab exposure for patients treated with pembrolizumab in the nonadjuvant setting at doses of 200 mg Q3W (*N* = 913) (a), 2 mg/kg Q3W (*N* = 542) (b), 10 mg/kg Q3W (*N* = 428) (c), 10 mg/kg Q2W (*N* = 117) (d). Figure includes ADA samples with corresponding PK concentrations. Samples taken >2 times the scheduled time were excluded. Individual pembrolizumab concentrations for the patients are represented as dots or crosses and mean value is represented by a black line. For the positive patients (non-TE and TE), all the samples (ADA negative and ADA positive) are “colored.” The confirmed positive ADA samples are indicated by a black circle around the corresponding PK sample. ADA, antidrug antibody; NAb, neutralizing antibody; non-TE, non–treatment-emergent ADA positive; PK, pharmacokinetic; Q2W, every 2 weeks; Q3W, every 3 weeks;

TE, treatment-emergent ADA positive.
