# Supplementary material for: Immunogenicity of pembrolizumab in patients with advanced tumors
Source: J Immunother Cancer. 2019 Aug 8;7:212. doi: 10.1186/s40425-019-0663-4 (PMC6686242; doi:10.1186/s40425-019-0663-4)

Additional file 5: **Figure S4** Pembrolizumab exposure for patients treated with pembrolizumab in the adjuvant setting at a dose of 200 mg Q3W (*N* = 495). Figure includes ADA samples with corresponding PK concentrations. Samples taken >2 times than the scheduled time were excluded. Individual pembrolizumab concentrations for the patients are represented as dots or crosses and mean value is represented by a black line. ADA, antidrug antibody; NAb, neutralizing antibody; non-TE, non–treatment-emergent ADA positive; Q3W, every 3 weeks; TE, treatment-emergent ADA positive.


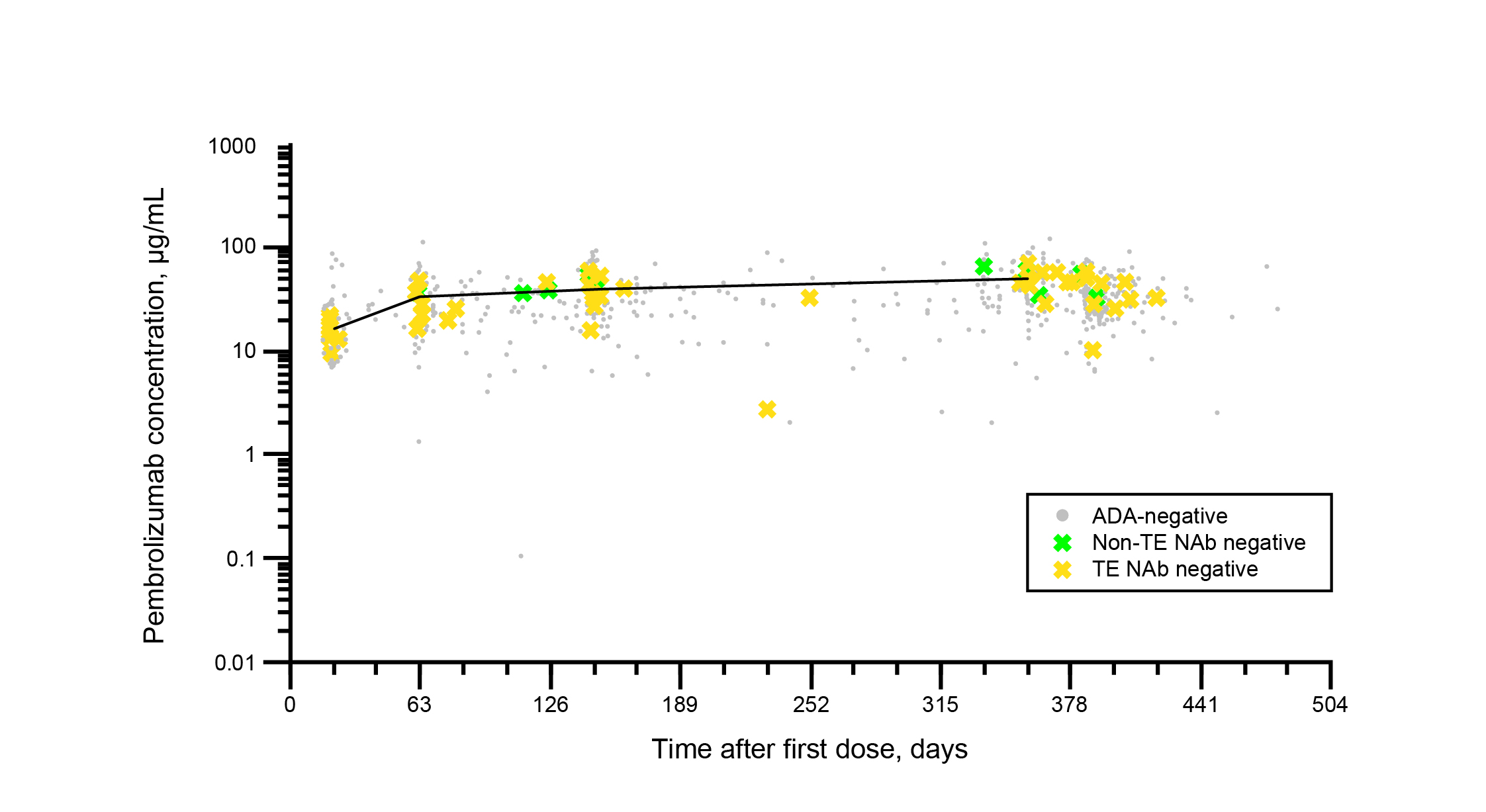

Supplement: Supplementary file 5 — Figure S4. Pembrolizumab exposure for patients treated with pembrolizumab in the adjuvant setting at a dose of 200 mg Q3W (N = 495). Figure includes ADA samples with corresponding PK concentrations. Samples taken > 2 times than the scheduled time were excluded. Individual pembrolizumab concentrations for the patients are represented as dots or crosses and mean value is represented by a black line. ADA, antidrug antibody; NAb, neutralizing antibody; non-TE, non–treatment-emergent ADA positive; PK, pharmacokinetic; Q3W, every 3 weeks; TE, treatment-emergent ADA positive. (DOCX 206 kb) [file 40425_2019_663_MOESM5_ESM.docx]
