# Supplementary material for: Immunogenicity of pembrolizumab in patients with advanced tumors
Source: J Immunother Cancer. 2019 Aug 8;7:212. doi: 10.1186/s40425-019-0663-4 (PMC6686242; doi:10.1186/s40425-019-0663-4)
Supplement: Supplementary file 6 — Table S1. Details of clinical studiesa included in the immunogenicity analysis of pembrolizumab. (DOCX 17 kb) [file 40425_2019_663_MOESM6_ESM.docx]

Additional file 6: **Table S1** Details of clinical studies^a^ included in the immunogenicity analysis of pembrolizumab

| **Indication** | **Study** | **ClinicalTrials.gov identifier** | **Data cutoff date** | **Study phase** | **Population** | **Patients** | |
| --- | --- | --- | --- | --- | --- | --- | --- |
|  |  |  |  |  |  | **Assessable^c^** | **Evaluable^d^** |
| Melanoma | KEYNOTE-001 (cohorts B and D) | NCT01295827 | 4/18/2014 | 1 | Advanced or metastatic melanoma | 617 | 125 |
|  | KEYNOTE-002 | NCT01704287 | 5/12/2014 | 2 | Advanced melanoma; ipilimumab-refractory | 311 | 120 |
|  | KEYNOTE-006 | NCT01866319 | 3/3/2015 | 3 | Advanced or metastatic melanoma with no prior systemic treatment (1st line) or one prior systemic treatment (2nd line) | 537 | 157 |
| NSCLC | KEYNOTE-001 (cohorts C and F) | NCT01295827 | 1/23/2015 | 1 | Advanced or metastatic NSCLC (treatment-naive or previously treated) | 483 | 136 |
|  | KEYNOTE-010 | NCT01905657 | 9/30/2015 | 2/3 | Previously treated, advanced NSCLC | 615 | 517 |
|  | KEYNOTE-024 | NCT02142738 | 5/9/2016 | 3 | Previously untreated, metastatic NSCLC | 138 | 138 |
| HNSCC | KEYNOTE-012 (cohort B) | NCT01848834 | 9/1/2015 | 1 | Advanced HNSCC | 56 | 17 |
|  | KEYNOTE-055  (first 50 patients) | NCT02255097 | 11/25/2015 | 2 | Platinum- and cetuximab-refractory recurrent or metastatic HNSCC | 45 | 45 |
| CRC  (MSI-high)^b^ | KEYNOTE-164 | NCT02460198 | 6/3/2016 | 2 | Previously treated advanced unresectable or metastatic MSI-H CRC | 54 | 54 |
| HL | KEYNOTE-013 | NCT01953692 | 5/27/2016 | 1 | Relapsed or refractory HL after brentuximab vedotin therapy | 23 | 4 |
|  | KEYNOTE-087 | NCT02453594 | 6/27/2016 | 2 | Relapsed or refractory HL | 197 | 178 |
| UC | KEYNOTE-012 (cohort C) | NCT01848834 | 9/1/2015 | 1 | Advanced or metastatic UC | 32 | 11 |
|  | KEYNOTE-045 | NCT02256436 | 9/7/2016 | 3 | Previously treated, recurrent or progressive metastatic UC | 240 | 231 |
|  | KEYNOTE -052 | NCT02335424 | 9/1/2016 | 2 | Cisplatin-ineligible, advanced and unresectable or metastatic UC | 307 | 267 |
| **Total** |  |  |  |  |  | **3655** | **2000** |

^a^KEYNOTE-054 (ClinicalTrials.gov identifier, NCT02362594) trial involving pembrolizumab in adjuvant setting is not included.

^b^All patients, except 1, were reported as MSI-high.

^c^Assessable patients were those with at least 1 postpembrolizumab dose sample with reportable ADA results.

^d^Evaluable patients were the total number of patients testing negative and positive for immunogenicity status (non–treatment emergent and treatment emergent).

ADA, antidrug antibody; CRC, colorectal carcinoma; HL, Hodgkin lymphoma; HNSCC, head and neck squamous cell carcinoma; MSI-H, microsatellite instability–high; NSCLC, non–small cell lung carcinoma; UC, urothelial cancer.
