# Supplementary material for: Immunogenicity of pembrolizumab in patients with advanced tumors
Source: J Immunother Cancer. 2019 Aug 8;7:212. doi: 10.1186/s40425-019-0663-4 (PMC6686242; doi:10.1186/s40425-019-0663-4)
Supplement: Supplementary file 7 — Table S2. Overview of pembrolizumab immunogenicity findings in KEYNOTE-054 (ClinicalTrials.gov Identifier, NCT02362594). (DOCX 13 kb) [file 40425_2019_663_MOESM7_ESM.docx]

Additional file 7: **Table S2**  Overview of pembrolizumab immunogenicity findings in KEYNOTE-054 (ClinicalTrials.gov Identifier, NCT02362594)

| **Pembrolizumab in the adjuvant setting: melanoma** | |
| --- | --- |
|  | **Treatment** |
| Immunogenicity status | 200 mg Q3W |
| Assessable patients^a^ | 496 |
| Inconclusive patients^b^ | 1 |
| Evaluable patients^c^ | 495 |
| Negative^d^ | 473 (95.6%) |
| Non–treatment-emergent positive^d^ | 5 (1.0%) |
| Neutralizing negative | 5 (1.0%) |
| Neutralizing positive | 0 |
| Treatment-emergent positive^d^ | 17 (3.4%) |
| Neutralizing negative | 17 (3.4%) |
| Neutralizing positive | 0 |

^a^Included are patients with at least 1 ADA sample available after treatment with pembrolizumab.

^b^Inconclusive patients are the number of patients with no positive ADA samples present and the drug concentration in the last sample above the DTL.

^c^Evaluable patients are the total number of negative and positive patients (non–treatment emergent and treatment emergent).

^d^Denominator was total number of evaluable patients.

ADA, antidrug antibody; DTL, drug tolerance limit; Q3W, every 3 weeks.
